# Knowledge, Attitude, and Practice Regarding Over-the-Counter Prescription of Topical Eye Medications Among the General Population of Saudi Arabia

**DOI:** 10.7759/cureus.80665

**Published:** 2025-03-16

**Authors:** Seham Al Hemaidi, Amjad Alharthi, Mohammed AlFehaid, Hussam Almalki, Yazeed A Hamzi, Abdulaziz Alnemari, Njood M Alhajri, Aljohrah M. Al Hunaif, Deena A Almaghrebi, Jehad Alabdulminaim

**Affiliations:** 1 Ophthalmology and Surgery, University of Tabuk, Tabuk, SAU; 2 College of Medicine, University of Tabuk, Tabuk, SAU; 3 College of Medicine, Prince Sattam Bin Abdulaziz University, Al Kharj, SAU; 4 College of Medicine, Umm Al-Qura University, Makkah, SAU; 5 College of Medicine, Jazan University, Jazan, SAU; 6 College of Medicine, Medicine and Surgery, Umm Al-Qura University, Makkah, SAU; 7 Ophthalmology, King Fahd University Hospital, Imam Abdulrahman Bin Faisal University, Dammam, SAU; 8 College of Medicine, King Khalid University, Abha, SAU; 9 Family Medicine, King Fahd University Hospital, Imam Abdulrahman Bin Faisal University, Dammam, SAU; 10 Ophthalmology, Zulfi General Hospital, Zulfi, SAU

**Keywords:** attitude, eye medications, over the counter, saudi arabia, topical knowledge

## Abstract

Background: Self-medication with over-the-counter (OTC) topical eye medications is prevalent and poses potential risks such as adverse effects, drug-drug interactions, and improper treatment. This study aimed to assess the knowledge, attitudes, and practices (KAP) of the general population in Saudi Arabia regarding the use of OTC topical eye medications.

Materials and methods: A descriptive cross-sectional study was conducted using an online Arabic questionnaire. A mixture of convenience and snowball sampling methods was employed to recruit participants. The target population included Saudi residents aged 18 and older. A total of 601 participants, comprising 324 males (53.9%) and 277 females (46.1%), were included. The questionnaire was retested for reliability before distribution. Data analysis was performed using SPSS version 26, with descriptive statistics and chi-square tests used to assess relationships between variables.

Results: The study found that 124 participants (91.9%) who used OTC eye drops once reported no adverse effects, while 11 participants (8.1%) experienced side effects. Among those who used OTC eye drops more frequently, 215 participants (98.6%) reported no adverse events, while three participants (1.4%) experienced adverse effects. A statistically significant relationship was found between the frequency of OTC eye drop use and the occurrence of side effects (p=0.005). Awareness of the risks associated with OTC eye drop misuse was generally low.

Conclusion: The findings highlight the need for targeted public health initiatives in Saudi Arabia to educate individuals about the safe and appropriate use of OTC topical eye medications and the potential risks of misuse.

## Introduction

Self-medication, particularly in the context of ophthalmic medications, is a widespread phenomenon with both beneficial and harmful implications. On one hand, self-medication allows individuals to access treatment quickly and conveniently, addressing common and minor health issues without the need for a medical consultation. On the other hand, it carries significant risks, particularly when used improperly [1,2]. The unsupervised use of medications, especially over-the-counter (OTC) drugs, can lead to serious health consequences, including adverse drug reactions, drug-drug interactions, and inappropriate management of medical conditions. In the case of ophthalmic self-medication, these risks include vision loss, prolonged disease progression, and the development of drug resistance [3,4].The practice of ophthalmic self-medication is observed to vary significantly around the world, with reported prevalence rates ranging from as low as 25.6% to as high as 73.6% [5-8]. This wide disparity highlights differing attitudes and access to eye care, as well as the varying levels of awareness and education regarding eye health among populations in different regions. In both developed and developing nations, the trend of using topical eye medications without a doctor's prescription has become prevalent [9,10]. However, there is a clear discrepancy between best practices and the current use of these OTC drugs. Many individuals are unaware of the proper dosage, indications, and potential side effects of these medications. This issue is particularly pronounced in countries where access to healthcare professionals may be limited or where there is a strong cultural inclination toward self-care [11,12]. In Saudi Arabia, the practice of self-medication is alarmingly common. A study by Al-Ghamdi et al. reported that approximately 81% of the general population has reported using OTC medications at some point in their lives [13]. This widespread use, while convenient for treating minor ailments, can also lead to serious consequences when not managed appropriately [14]. In the case of ophthalmic medications, improper use can result in microbial resistance, suboptimal treatment outcomes, adverse reactions, and the progression of eye diseases [15]. Additionally, many individuals may not fully understand the potential risks associated with using OTC eye medications, such as allergic reactions, toxicity, or the worsening of underlying conditions like glaucoma or dry eye syndrome [16]. The misuse of OTC ophthalmic medications presents a significant concern due to the delicate nature of the eye and the potential for irreversible damage. Inappropriate use of these medications can manifest in various ways, including using eye drops intended for allergies to self-treat infections, potentially exacerbating the condition [9]. Additionally, unsupervised use of OTC eye medications raises concerns about potential drug-drug interactions, particularly for individuals concurrently taking prescribed medications for other health conditions [17]. Such interactions may lead to reduced efficacy or an increased risk of adverse effects. Furthermore, self-treating with OTC ophthalmic medications can mask the symptoms of more serious underlying eye conditions, leading to delayed diagnosis and appropriate treatment [18]. This delay can have significant consequences, potentially resulting in irreversible vision loss. The increasing trend of ophthalmic self-medication highlights the need for public health interventions that promote the safe and appropriate use of OTC topical eye medications. Therefore, understanding the factors influencing self-medication practices with ophthalmic medications is crucial. A key aspect is assessing the knowledge, attitudes, and behaviors of individuals who rely on OTC ophthalmic medications. Studies have shown that a lack of awareness regarding proper eye drop usage, potential side effects, and the importance of consulting a healthcare professional contributes to misuse [16,19,20]. Furthermore, misconceptions about the safety and efficacy of OTC eye medications, often fueled by inadequate health literacy or misleading marketing, can lead to inappropriate self-treatment [21]. Addressing these knowledge gaps and promoting responsible use of OTC ophthalmic medications through public health campaigns and educational initiatives is essential to minimize potential harm. These campaigns could focus on educating the public about the risks of unsupervised eye medication use, the importance of consulting a healthcare professional before using eye drops or ointments, and recognizing the signs of adverse reactions. Furthermore, healthcare professionals themselves play a crucial role in addressing this issue. Pharmacists and physicians should provide better counseling and education to individuals seeking OTC eye medications, ensuring that they understand the correct indications, dosage, and potential risks of the drugs. By fostering a more informed and cautious approach to self-medication, the incidence of adverse effects, such as microbial resistance and visual morbidity, can be reduced. This study was, therefore, essential for public health initiatives in Saudi Arabia, as it aims to bridge the knowledge gap regarding the use of OTC topical eye medications. By investigating the knowledge, attitudes, and practices (KAP) of the general population, this research can inform healthcare strategies designed to promote safe self-medication practices.

## Materials and methods

This descriptive cross-sectional study was conducted to assess the KAP of the general population in Saudi Arabia regarding the use of OTC topical eye medications. The study targeted individuals over the age of 18 who were residing in Saudi Arabia during the data collection period. The methodology was designed to provide a representative snapshot of the population's behavior and awareness concerning self-medication with ophthalmic drugs.

Study population and sampling method

The target population for this study included both male and female adults living in the Kingdom of Saudi Arabia. The inclusion criteria were individuals aged 18 years and older, irrespective of their educational or socio-economic background. The exclusion criteria were non-residents of Saudi Arabia and individuals below the age of 18. A simple random sampling method was employed to ensure a fair and unbiased selection of participants. This method was chosen to minimize sampling bias and ensure that every individual within the target population had an equal chance of being selected. The calculated sample size for this study was 385 participants, determined using a standard sample size calculation formula to achieve sufficient statistical power. However, to account for potential data loss due to incomplete responses or dropout, the sample size was increased to 600 participants. This larger sample size also allowed for an equal gender distribution, aiming for 300 male and 300 female participants to ensure that the study captured gender-specific trends in the use of OTC topical eye medications.

Questionnaire design

Data were collected using a structured online questionnaire, which was developed in Arabic to ensure accessibility and comprehension for the Saudi population. The questionnaire was designed using Google Forms, an accessible and user-friendly platform for online surveys. It was adapted from a previously used questionnaire in a prior study to ensure reliability and validity. The questions were carefully crafted to assess the respondents' KAP regarding the use of OTC topical eye medications.

The questionnaire was divided into two sections. The first section, demographics, collected information on participants' age, gender, educational level, and region of residence. The second section, self-medication with topical eye drops: usage, knowledge, and attitudes, assessed participants' awareness of the ingredients, indications, and side effects of commonly used over-the-counter (OTC) eye drops. It also evaluated their perceptions of the importance of consulting an ophthalmologist before using these medications and their overall attitudes toward self-medication. Additionally, this section explored the frequency and types of OTC eye drops used, as well as any experiences with adverse reactions related to their use.

The questionnaire was pretested on a small group of individuals (n=20) to ensure clarity, reliability, and validity of the questions. Minor modifications were made based on feedback from the pilot test to improve the quality of the data collection tool.

Data collection procedure

Data were collected over a period of several weeks, starting on September 19, 2024, and concluding on October 20, 2024. An online distribution strategy was employed, with respondents invited to participate in the survey through electronic links shared via social media platforms, emails, and messaging apps. The links were accompanied by a brief explanation of the study’s objectives, the target population, and a voluntary participation request. Participants were informed that their responses would be anonymous and used solely for research purposes. Informed consent was obtained electronically before respondents proceeded to answer the questionnaire. To maximize the response rate and ensure that the gender balance was maintained, additional efforts were made to reach a diverse group of participants across different regions of Saudi Arabia. Reminders were sent periodically to encourage participation and ensure that the target sample size of 600 participants was reached.

Ethical considerations

This study adhered to the ethical principles outlined in the Declaration of Helsinki. Participation in the survey was entirely voluntary, and participants were informed of their right to withdraw from the study at any time. Informed consent was obtained from all participants before they began the survey, and confidentiality was strictly maintained throughout the study. No personal identifiers were collected, and the data were securely stored and accessible only to the research team. Ethical approval for the study was obtained from the relevant institutional review board before the commencement of the research.

Data analysis

Once data collection was completed, the responses were exported from Google Forms into Microsoft Excel and subsequently imported into SPSS version 26 (IBM Corp., Armonk, NY, USA). Descriptive statistics were used to summarize the demographic characteristics of the participants and to analyze the variables related to their KAP regarding OTC topical eye medications. Frequencies and percentages were calculated for categorical variables, while means and standard deviations were reported for continuous variables. Cross-tabulation and chi-square tests were employed to examine the associations between different variables, such as gender, educational level, and awareness of the risks associated with the use of OTC topical eye medications. A significance level of p<0.05 was set to determine statistically significant associations.

## Results

The study included 601 participants from Saudi Arabia, with 53.9% male and 46.1% female. The majority (56.9%) were aged between 18-30 years, followed by 33.1% aged 31-50 years, 9.3% aged 51-70 years, and 0.7% over 70 years. Most participants (93.5%) were Saudi nationals, while 6.5% were non-Saudi. Regarding the province of residence, 41.9% lived in the Western region, 20.3% in the Southern region, 17.5% in the Central region, 13.0% in the Eastern region, and 7.3% in the Northern region. In terms of education, 44.4% had a university degree, 44.1% completed high school, 4.3% attended primary school, 2.7% attended middle school, 2.5% had a Master's or PhD, and 2.0% were uneducated (Table 1).

**Table 1:** Sociodemographic details

Sociodemographic parameters		N	%
Gender	Female	277	46.1
	Male	324	53.9
Age	18-30 years	342	56.9
	31-50 years	199	33.1
	51-70 years	56	9.3
	>70 years	4	.7
Nationality	Saudi	562	93.5
	Non-Saudi	39	6.5
Province of residence in Saudi Arabia	Western	252	41.9
	Southern	122	20.3
	Central	105	17.5
	Eastern	78	13.0
	Northern	44	7.3
Educational level	Primary school	26	4.3
	Middle school	16	2.7
	High school	265	44.1
	University	267	44.4
	Master's or PhD	15	2.5
	Uneducated	12	2.0

The analysis of practices related to eye drops is shown in Table 2. The survey revealed that 359 (59.7%) participants had used eye drops or ointments not prescribed by a doctor. Among the 359 individuals who reported such usage, the most common reasons for not consulting an ophthalmologist included feeling qualified to choose their own medication (21.8%), lack of time (17.8%), having medication at home (7.8%), and recommendations from friends or family (11.1%). The majority (76.0%) used sterilizers or moisturizers, while only a small percentage opted for anti-allergic (5.8%) or antibiotic drops (2.5%). A significant number (82.7%) believed it was very important to visit an ophthalmologist before obtaining eye drops. Regarding the frequency of over-the-counter eye drop usage, 60.7% used them more than once, with 48.2% stating they were responsible for their own prescriptions. The primary reasons for using eye drops included dryness (59.6%) and itchy eyes (14.8%). At the same time, it was found that 56.8% of participants reported reading the leaflet that accompanied the medicine before use.

**Table 2:** Practices related to eye drops

		N	%
Ever used eye drops/ointment that were not prescribed by your doctor	No	242	40.3
	Yes	359	59.7
Reasons for not going to a doctor. Ophthalmologist for eye drops (N=359)	I consider myself qualified to know which medication to use	78	21.8
	I didn't have time to go to the doctor	64	17.8
	I had medicine at home	28	7.8
	I didn't have the money to pay for the medical consultation	14	3.9
	The eye drops calmed my symptoms	42	11.7
	The pharmacist recommended it to me	56	15.6
	This medicine was recommended by a friend/family	40	11.1
	I couldn't stand my symptoms	7	1.9
	I didn't have the money to buy the medicine I was prescribed	1	.3
	I was out of town	2	.6
	I was trying to avoid going to the doctor	9	2.5
	The eye drops were discounted and on sale	1	.3
	Other unspecified causes	17	4.7
Medications used (n=359)	Sterilizer/moisturizer	273	76.0
	Anti-allergic	21	5.8
	Home remedies	22	6.1
	Cortisone	11	3.1
	Antibiotic	9	2.5
	Antibiotic plus vasoconstrictor drops	1	.3
	Vasoconstrictive drops	2	.6
	Vasoconstrictor drops plus NSAIDs	3	.8
	I don't remember	17	4.7
Importance of visiting an ophthalmologist before getting eye drops	Unimportant	59	16.4
	Very important	297	82.7
	Not sure	3	.8
Frequency of using over counter eye drops (N=359)	Once	135	37.6
	More than once	218	60.7
	Not sure/don’t remember	6	1.7
Person responsible for prescribing eye drops (N=359)	Myself	173	48.2
	My family	61	17.0
	My friends	29	8.1
	Pharmacist	80	22.3
	Social media sites	16	4.5
Reason for using (N=359)	Dryness	214	59.6
	Eye discharge	12	3.3
	Eye pain	28	7.8
	Eye redness	52	14.5
	Itchy eyes	53	14.8
Read the leaflet that came with the medicine before using it (n=359)	No	155	43.2
	Yes	204	56.8

The analysis of the relationship between the use of eye drops or ointments that were not prescribed by a doctor and various sociodemographic factors among 359 participants is given in Table 3. Gender differences showed that a higher percentage of females (63.9%) reported using unprescribed eye drops compared to males (56.2%), although the p-value of 0.054 indicates this was not statistically significant. Age did not significantly influence usage, with similar percentages across age groups. Nationality was also not significantly associated, as 60.7% of Saudi participants and 46.2% of non-Saudis reported using unprescribed drops (p=0.074). However, significant regional variations were noted: 63.9% of individuals from the Western region reported using unprescribed drops (p=0.028). Education level was the most influential factor, with 80.8% of primary school graduates and 41.5% of high school graduates having never used unprescribed eye drops (p=0.001), indicating a clear trend where higher education correlates with greater usage of these medications without a prescription.

**Table 3:** Relationship between usage of eye drops/ointment that were not prescribed by a doctor and sociodemographic details (n=601) Statistical analysis was performed using Pearson's chi-square test.

Sociodemographic details		Used or have you ever used eye drops/ointment that were not prescribed by a doctor		p-value
		No (n=242)	Yes (n=359)	
Gender	Female	100 (36.1%)	177 (63.9%)	0.054
	Male	142 (43.8%)	182 (56.2%)	
Age	18-30 years	133 (38.9%)	209 (61.1%)	0.610
	31-50 years	87 (43.7%)	112 (56.3%)	
	51-70 years	21 (37.5%)	35 (62.5%)	
	>70 years	1 (25.0%)	3 (75.0%)	
Nationality	Saudi	221 (39.3%)	341 (60.7%)	0.074
	Non-Saudi	21 (53.8%)	18 (46.2%)	
Place of residence	Western	91 (36.1%)	161 (63.9%)	0.028
	Southern	47 (38.5%)	75 (61.5%)	
	Central	57 (54.3%)	48 (45.7%)	
	Eastern	29 (37.2%)	49 (62.8%)	
	Northern	18 (40.9%)	26 (59.1%)	
Educational level	Primary school	21 (80.8%)	5 (19.2%)	0.001
	Middle school	6 (37.5%)	10 (62.5%)	
	High school	110 (41.5%)	155 (58.5%)	
	University	95 (35.6%)	172 (64.4%)	
	Master’s or PhD	5 (33.3%)	10 (66.7%)	
	Uneducated	5 (41.7%)	7 (58.3%)	

The assessment of knowledge and experience regarding eye drops among 359 participants is given in Table 4. It was observed that about 53.5% of respondents reported not knowing the ingredients of the eye drops they used and about 52.6% of participants were unaware of the side effects associated with the eye drops. Despite this lack of knowledge, the vast majority (96.1%) reported not experiencing any adverse events from using the drops, with only 3.9% stating they had encountered side effects.

**Table 4:** Knowledge and experience regarding the eye drops (n=359)

Knowledge and experience		N	%
Know the ingredients of the medicine used	No	192	53.5
	Yes	167	46.5
Know about the side effects of eye drops used	No	189	52.6
	Yes	170	47.4
Experienced any adverse events from using the drops	No	345	96.1
	Yes	14	3.9

The importance of visiting an ophthalmologist before using eye drops was perceived differently based on education (p=0.023). None (0.0%) of the primary school participants deemed it unimportant, while 1.7% of middle school, 45.8% of high school, 42.4% of university, 3.4% of master’s or PhD, and 6.8% of the uneducated felt similarly. A higher percentage of individuals with a university education considered it very important, reflecting that education correlates with the perceived necessity of professional advice. Awareness of the side effects of eye drops showed a significant relationship with educational level, where higher educational attainment was associated with better awareness of side effects (p=0.049). Only 1.1% of participants with a primary school education were unaware of side effects, while 4.2% of those with a middle school education, 44.4% with high school, 43.9% with university, 2.6% with master’s or PhD, and 3.7% of the uneducated were not aware. However, no statistically significant differences were observed across educational levels (p<0.05) in the frequency of using OTC eye drops, who encouraged the use of non-prescription drugs, the practice of reading the leaflet that accompanies the medication, and knowledge regarding the ingredients of the medicine used (Table 5).

**Table 5:** Relationship between educational level and practices, knowledge, and perceptions related to eye drop use Statistical analysis was performed using Pearson's chi-square test.

Practice/knowledge/perception		Educational level						P-value
		Primary school	Middle school	High school	University	Master's or PhD	Uneducated	
Importance of visiting an ophthalmologist before getting eye drops	Unimportant	0 (0.0%)	1 (1.7%)	27 (45.8%)	25 (42.4%)	2 (3.4%)	4 (6.8%)	0.023
	Very important	5 (1.7%)	8 (2.7%)	127 (42.8%)	146 (49.2%)	8 (2.7%)	3 (1.0%)	
	Not sure	0 (0.0%)	1 (33.3%)	1 (33.3%)	1 (33.3%)	0 (0.0%)	0 (0.0%)	
Frequency of usage of over-the-counter eye drops	Once	2 (1.5%)	5 (3.7%)	55 (40.7%)	70 (51.9%)	2 (1.5%)	1 (0.7%)	0.322
	More than once	3 (1.4%)	5 (2.3%)	98 (45.0%)	99 (45.4%)	8 (3.7%)	5 (2.3%)	
	Not sure	0 (0.0%)	0 (0.0%)	2 (33.3%)	3 (50.0%)	0 (0.0%)	1 (16.7%)	
Person encouraged to use non-prescription drugs	My family	1 (1.6%)	1 (1.6%)	32 (52.5%)	23 (37.7%)	2 (3.3%)	2 (3.3%)	0.843
	My friends	0 (0.0%)	2 (6.9%)	11 (37.9%)	15 (51.7%)	0 (0.0%)	1 (3.4%)	
	Myself	4 (2.3%)	5 (2.9%)	73 (42.4%)	83 (48.3%)	5 (2.9%)	2 (1.2%)	
	Pharmacist	0 (0.0%)	1 (1.3%)	36 (45.0%)	40 (50.0%)	2 (2.5%)	1 (1.3%)	
	Social media sites	0 (0.0%)	1 (6.3%)	3 (18.8%)	10 (62.5%)	1 (6.3%)	1 (6.3%)	
Read the leaflet that came with the medicine before using it	No	1 (0.6%)	5 (3.2%)	68 (43.9%)	71 (45.8%)	6 (3.9%)	4 (2.6%)	0.657
	Yes	4 (2.0%)	5 (2.5%)	87 (42.6%)	101 (49.5%)	4 (2.0%)	3 (1.5%)	
Know the ingredients of the medicine used	No	2 (1.0%)	7 (3.6%)	77 (40.1%)	96 (50.0%)	6 (3.1%)	4 (2.1%)	0.708
	Yes	3 (1.8%)	3 (1.8%)	78 (46.7%)	76 (45.5%)	4 (2.4%)	3 (1.8%)	
Aware of the side effects of the eye drops used	No	2 (1.1%)	8 (4.2%)	84 (44.4%)	83 (43.9%)	5 (2.6%)	7 (3.7%)	0.049
	Yes	3 (1.8%)	2 (1.2%)	71 (41.8%)	89 (52.4%)	5 (2.9%)	0 (0.0%)	

The relationship between the frequency of using OTC eye drops and the experience of adverse events shows that among participants who used eye drops once, 91.9% reported no adverse events, while 8.1% experienced side effects. In contrast, those who used eye drops more than once had a much lower rate of side effects, with 98.6% reporting no adverse events and only 1.4% experiencing side effects (p=0.005) (Table 6).

**Table 6:** Relationship between frequency of usage and experience of side effects Statistical analysis was performed using Pearson's chi-square test.

Frequency of using over-the-counter eye drops	Experienced adverse events		P-value
	No	Yes	
Once	124 (91.9%)	11 (8.1%)	0.005
More than once	215 (98.6%)	3 (1.4%)	
Not sure	6 (100.0%)	0 (0.0%)	

## Discussion

Our study revealed a concerning prevalence of self-medication practices with ophthalmic medications among the surveyed population. Over half of the participants (59.7%) reported using eye drops or ointments without a prescription from an ophthalmologist. This finding aligns with previous studies highlighting the widespread nature of self-medication with ophthalmic medications globally [16,18,22]. The most common reasons cited for not consulting an ophthalmologist were feeling qualified to choose their own medication, lack of time, having medication readily available at home, and recommendations from friends or family. Studies highlighted a significant gap between best practices and the current use of OTC medications, urging a powerful call for educational initiatives to transform this behavior [23,24]. In Saudi Arabia, where OTC drug consumption is regulated, studies have shown that a significant percentage of the population irrespective of age and gender has embraced these medications at some stage in their lives [25-27]. However, the misuse of ophthalmic OTC products poses serious risks, including microbial resistance and suboptimal treatment outcomes, which can impede health and progress. These findings underscore the need for targeted educational interventions to address misconceptions about self-treating eye conditions and emphasize the importance of seeking professional medical advice [28]. While OTC is readily available, ophthalmic medications can have adverse effects, interact with other medications, and potentially delay the diagnosis and treatment of more serious eye conditions [17]. Interestingly, despite a significant proportion engaging in self-medication, the majority of participants (82.7%) acknowledged the importance of consulting an ophthalmologist before using eye drops. This discrepancy could indicate a knowledge-behavior gap, where individuals understand the ideal course of action but face barriers to implementing it, such as a perceived lack of time or access to healthcare [29]. The high percentage of participants using moisturizers (76%) suggests that dryness is a common complaint prompting self-medication. However, it is crucial to differentiate simple dryness from more serious conditions that require specific treatment [30]. The use of anti-allergic and antibiotic drops, although less frequent, further highlights the potential risks associated with self-medication. Using these medications without proper diagnosis can lead to ineffective treatment, potential side effects, and the emergence of antibiotic resistance [31]. The finding that 56.8% of participants reported reading the leaflet accompanying the medication is encouraging. However, it also underscores the importance of clear and accessible information on these leaflets. Efforts should be made to ensure that information regarding potential risks, contraindications, and when to seek professional medical advice is prominently displayed and easily understandable [32]. Interestingly, although a higher percentage of females (63.9%) reported using unprescribed eye drops compared to males (56.2%), this difference was not statistically significant. This finding suggests that gender, in isolation, may not be a strong predictor of self-medication with ophthalmic medications [1,33]. Similarly, age and nationality did not show a statistically significant association with the use of unprescribed eye drops. However, our study did identify significant regional variations. Individuals from the Western region were significantly more likely to report using unprescribed eye drops (63.9%). This finding highlights the potential influence of regional factors, such as access to healthcare, cultural beliefs, and socioeconomic factors, on self-medication practices [34,35]. Further investigation into the specific factors contributing to this regional variation is warranted. The most striking finding was the significant association between education level and the use of unprescribed eye drops. Our study demonstrated a clear trend where higher education levels correlated with a greater likelihood of using these medications without a prescription. Specifically, 80.8% of primary school graduates and 41.5% of high school graduates reported never having used unprescribed eye drops, compared to a significantly lower percentage among those with higher education levels. This finding contradicts the common assumption that higher health literacy, often associated with higher education levels, would lead to a decreased likelihood of engaging in risky health behaviors like self-medication [36,37]. Our study revealed a concerning disconnect between participants' knowledge about ophthalmic medications and their reported experiences of side effects. Despite a significant lack of knowledge regarding ingredients and potential side effects, the vast majority of participants (96.1%) reported not experiencing any adverse events. This finding raises several important points for consideration. First, the self-reported nature of side effects may lead to underreporting. Research shows that people may not attribute certain symptoms to the eye drops, especially if they are mild or non-specific [38]. Additionally, recall bias may come into play, with participants potentially forgetting past experiences of side effects [39]. Second, the lack of reported side effects does not necessarily equate to the absence of risk. Some side effects may be delayed or subtle, making it difficult for individuals to establish a causal link with the eye drops [32]. Moreover, certain individuals may be more susceptible to specific side effects based on factors such as age, underlying health conditions, or concurrent use of other medications [40]. Third, the high percentage of participants unaware of potential side effects (52.6%) highlights a critical gap in knowledge. This lack of awareness can have significant implications, potentially leading to inappropriate self-treatment, delayed medical attention when side effects do occur, and an increased risk of complications [41]. It is also crucial to consider that the most commonly used eye drops in our study were sterilizers and moisturizers (76%). These types of eye drops are generally associated with a lower risk of serious side effects compared to, for example, antibiotic or steroid eye drops [32,42]. This factor may partially explain the low reported rate of side effects in our study.

Our study revealed an intriguing and statistically significant (p=0.005) inverse relationship between the frequency of OTC eye drop use and the reported experience of adverse events. While 8.1% of participants who used eye drops once reported side effects, this rate decreased to 1.4% among those using drops more frequently. This finding contradicts the general expectation that increased exposure to medication, even OTC, would correlate with a higher risk of adverse events [43]. Participants using eye drops only once might have a more vivid memory of any adverse events, even if minor and transient, compared to those using drops more regularly [19]. This could lead to an overestimation of side effects in the "once" group. Furthermore, the study relied solely on self-reported adverse events without objective verification. Participants might not attribute certain symptoms to the eye drops, especially if mild or non-specific, potentially leading to underreporting in both groups [38]. Additionally, the study did not differentiate between types of OTC eye drops used, which could vary significantly in their ingredient profiles and potential side effect profiles [44].

This study has some limitations. First, most of our study participants were aged between 18 and 30 years and from the western region, which may impact the generalizability of the results. Another limitation that might affect generalizability is that the online self-administered questionnaire was limited to people with access to the internet. Furthermore, data about OTC prescription of topical eye medications were self-reported, which might introduce recall or social desirability bias.

## Conclusions

This study revealed a high prevalence of unprescribed eye drop usage, with 59.7% of participants engaging in this practice. Significant regional differences were found, particularly in the Western region, and educational level was the strongest predictor, with higher education correlating with greater usage and better awareness of side effects (p=0.049). Importantly, the perceived necessity of consulting an ophthalmologist was strongly associated with education. Despite frequent use, 96.1% of participants reported no adverse events. Targeted public health campaigns are needed to raise awareness about the risks of using unprescribed eye drops, particularly among less-educated populations. The importance of consulting healthcare professionals before using eye medication should reassure our audience about the safety and reliability of the healthcare system. Further research could explore the long-term effects of unprescribed eye drop use and the development of educational tools to improve safe medication practices.

## Appendices

Questionnaire

Section A: Demographic Information

1. Gender:

Male

Female

2. Age:

18-30

31-50

51-70

Older than 70

3. Region of Residence:

Western

Eastern

Northern

Southern

Central

4. Educational Level:

Primary school

Middle school

High school

University degree

Master’s or PhD

Not educated

5. Nationality:

Saudi

Non-Saudi

Section B: Knowledge, Attitudes, and Practices Regarding OTC Eye Medications

6. Have you ever used eye drops or ointments that were not prescribed by a doctor?

Yes

No

7. If yes, which type of medication did you use?

Sterilizer/moisturizer

Anti-allergic

Home remedies

Cortisone

Antibiotic

Vasoconstrictive drops

Vasoconstrictive drops plus NSAIDs

Antibiotics plus vasoconstrictive agent

I don’t remember the medication

8. How many times have you used OTC eye drops without a prescription?

Once

More than once

Not sure/don’t remember

9. Who encouraged you to use non-prescribed eye drops?

Myself

Family

Friends

Pharmacist

Social media

10. What was your primary reason for using OTC eye drops?

Dryness

Eye redness

Itchy eyes

Eye discharge

Eye pain

11. What was your primary reason for not consulting an ophthalmologist before using OTC eye drops?

I consider myself qualified to know which medication to use

A friend or family member recommended the medication

I didn’t have time to visit the physician

I was trying to avoid visiting the physician

I had no money to pay for a medical consultation

I already had the medication at home

The pharmacist recommended it to me

I was out of town

I couldn’t tolerate my symptoms

I didn’t have money to buy the prescribed medicine

The eye drops were on sale

The eye drops relieved my symptoms

Other (please specify)

12. How important do you think it is to visit an ophthalmologist before obtaining eye drops?

Very important

Not important

Not sure

13. Do you know the ingredients of the eye drops you use?

Yes

No

14. Are you aware of the potential side effects of the eye drops you use?

Yes

No

15. Have you read the patient information leaflet before using the eye drops?

Yes

No

16. Have you ever experienced any adverse effects from using OTC eye drops?

Yes

No

17. If yes, please specify: (open-ended response)
